# 2-(Morpholin-4-yl)-6-(1*H*-pyrrol-1-yl)­pyridine-3,5-dicarbonitrile

**DOI:** 10.1107/S160053681200815X

**Published:** 2012-02-29

**Authors:** Peter N. Horton, Shaaban K. Mohamed, Ahmed M. Soliman, Eman M. M. Abdel-Raheem, Mehmet Akkurt

**Affiliations:** a School of Chemistry, University of Southampton, Highfield, Southampton SO17 1BJ, England; b Chemistry and Environmental Science Division, School of Science, Manchester Metropolitan University, England; c Department of Chemistry, Faculty of Science, Sohag University, Sohag, Egypt; d Department of Physics, Faculty of Sciences, Erciyes University, 38039 Kayseri, Turkey

## Abstract

In the title compound, C_15_H_13_N_5_O, the morpholine ring adopts a chair conformation. The dihedral angle between the pyrrole ring and the pyridine ring is 28.93 (14)°. In the crystal, the molecules are linked by C—H⋯O hydrogen bonds occur, and aromatic weak π–π stacking [centroid–centroid separation = 4.178 (2) Å] and C—H⋯π inter­actions consolidate the packing.

## Related literature

For the biological activity of pyridine derivatives, see: Altomare *et al.* (2000 ▶); Basavaraja *et al.* (2010 ▶); Cho *et al.* (2001 ▶); Goda *et al.* (2004 ▶); Hosni & Abdualla (2008 ▶); Kovala-Demertzi *et al.* (2007 ▶); Mikail *et al.* (2001 ▶); Sylvie *et al.* (2002 ▶); Tiwari *et al.* (2002 ▶); Yeong *et al.* (2004 ▶). For the definition of puckering parameters, see: Cremer & Pople (1975 ▶).
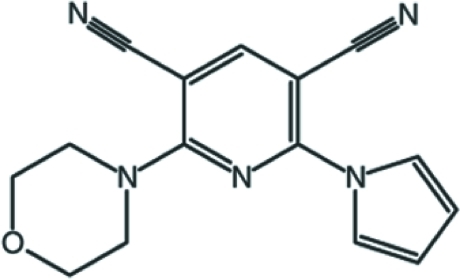


## Experimental

### 

#### Crystal data

C_15_H_13_N_5_O
*M* *_r_* = 279.30
Triclinic, 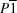

*a* = 8.633 (2) Å
*b* = 8.763 (3) Å
*c* = 9.559 (3) Å
α = 91.715 (7)°
β = 108.110 (8)°
γ = 100.572 (7)°
*V* = 672.7 (4) Å^3^
*Z* = 2
Mo *K*α radiation
μ = 0.09 mm^−1^
*T* = 120 K
0.52 × 0.44 × 0.18 mm

#### Data collection

Rigaku R-AXIS conversion diffractometer
Absorption correction: multi-scan (*CrystalClear-SM Expert*; Rigaku, 2011 ▶) *T*_min_ = 0.953, *T*_max_ = 0.984
8461 measured reflections
3067 independent reflections
1884 reflections with *I* > 2σ(*I*)
*R*_int_ = 0.081

#### Refinement

*R*[*F* ^2^ > 2σ(*F* ^2^)] = 0.075
*wR*(*F* ^2^) = 0.225
*S* = 1.05
3067 reflections
190 parameters
H-atom parameters constrained
Δρ_max_ = 0.35 e Å^−3^
Δρ_min_ = −0.39 e Å^−3^

### 

Data collection: *CrystalClear-SM Expert* (Rigaku, 2011 ▶); cell refinement: *CrystalClear-SM Expert*; data reduction: *CrystalClear-SM Expert*; program(s) used to solve structure: *SHELXS97* (Sheldrick, 2008 ▶); program(s) used to refine structure: *SHELXL97* (Sheldrick, 2008 ▶); molecular graphics: *ORTEP-3 for Windows* (Farrugia, 1997 ▶) and *PLATON* (Spek, 2009 ▶); software used to prepare material for publication: *WinGX* (Farrugia, 1999 ▶) and *PLATON*.

## Supplementary Material

Crystal structure: contains datablock(s) global, I. DOI: 10.1107/S160053681200815X/hg5182sup1.cif

Structure factors: contains datablock(s) I. DOI: 10.1107/S160053681200815X/hg5182Isup2.hkl

Supplementary material file. DOI: 10.1107/S160053681200815X/hg5182Isup3.cml

Additional supplementary materials:  crystallographic information; 3D view; checkCIF report

## Figures and Tables

**Table 1:** Hydrogen-bond geometry (Å, °) *Cg*3 is the centroid of the N1/C1–C5 pyridine ring.

*D*—H⋯*A*	*D*—H	H⋯*A*	*D*⋯*A*	*D*—H⋯*A*
C8—H8⋯O1^i^	0.93	2.44	3.294 (3)	152
C12—H12*B*⋯O1^ii^	0.97	2.51	3.397 (3)	153
C12—H12*A*⋯*Cg*3^iii^	0.97	2.92	3.429 (3)	114

Symmetry codes: (i) 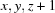; (ii) 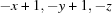; (iii) 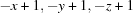.

**Fig. 1.**
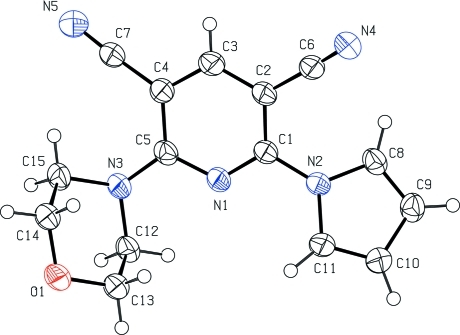
The molecular structure of (I), showing the atom-numbering scheme and displacement ellipsoids drawn at the 50% probability level.

**Fig. 2.**
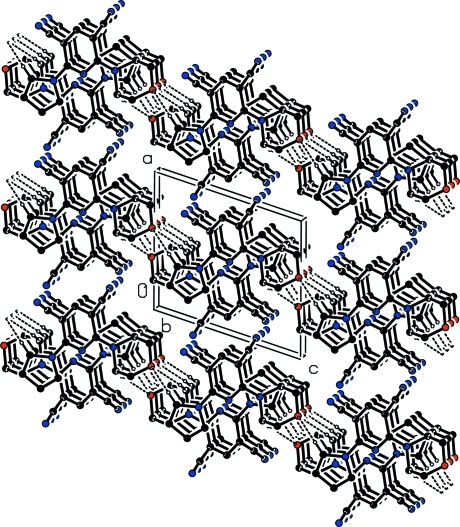
View of the packing and hydrogen bonding of (I) down the b axis. The hydrogen atoms not involved in the hydrogen bonds have been omitted for clarity.

## supplementary crystallographic information

### Comment

The pyridine skeleton is of great importance to chemists as well as to biologists as it is found in a large variety of naturally occurring compounds and also in clinically useful molecules having diverse biological activities. Its derivatives are known to possess antitubercular (Mikail *et al.*, 2001), anti-ulcer (Cho *et al.*, 2001), antimicrobial (Yeong *et al.*, 2004; Goda *et al.*, 2004), antitumor (Tiwari *et al.*, 2002; Kovala-Demertzi *et al.*, 2007), antiviral (Sylvie *et al.*, 2002) and cardio tonic properties (Altomare *et al.*, 2000). Poly-substituted pyridines, especially the 3,5-pyridinedicarbonitriles, are interesting as antioxidants and NADH co-enzyme analogues that mediate hydrogen transfer in biological systems, and for their antihistaminic, anti-inflammatory and analgesic activity (Hosni & Abdualla, 2008). In addition, it was found that drugs containing morpholine moiety in their structures have exhibited remarkable biological properties (Basavaraja *et al.*, 2010). These facts stimulated us to synthesis the title compound for its potential biological activity.

The title molecule (I) has an open conformation as shown in Fig. 1. The N3/O1/C12–C15 morpholine ring adopts a chair conformation [puckering parameters (Cremer & Pople, 1975): Q_T_ = 0.576 (3) Å, θ = 3.0 (3) ° and φ = 131 (6) °]. The pyridine ring is almost planar with maximum deviations of -0.061 (3) Å for C2 and -0.69 (3) Å for C5 [puckering parameters: Q_T_ = 0.117 (3) Å, θ = 86.4 (15) ° and φ = 283.7 (13) °]. The N1/C1–C5 pyridine ring makes a dihedral angle of 28.93 (14)° with the N2/C8–C11 pyrrole ring which is essentially planar with a maximum deviation of 0.009 (3) Å for C11.

The crystal structure is stabilized by intermolecular C—H···O hydrogen bonds (Table 1, Fig. 2) and weak π-π stacking [*Cg*1···*Cg*3(1 - *x*, -*y*, 1 - *z*) = 4.178 (2) Å; where *Cg*1 and *Cg*3 are the centroids of the N2/C8–C11 pyrrole and N1/C1–C5 pyridine rings, respectively] and C—H···π interactions.

### Experimental

An equimolar mixture of 2-amino-6-morpholin-4-ylpyridine-3,5-dicarbonitrile and 2,5-dimethoxytetrahydrofuran was refluxed in acetic acid at 491 K for one hour. The solid was obtained on cooling, filtered, washed with water and re-crystallized from ethanol to afford the title compound. 89% yield, m.p. 433 K. Needle crystals of the title compound, suitable for X-ray diffraction, were obtained by slow evaporation of a solution in ethanol over 24 h.

### Refinement

All H-atoms were placed in calculated positions [C—H = 0.93 Å for aromatic and C—H = 0.97 Å for methylene *U*_iso_(H) = 1.2 *U*_eq_(C)] and were refined using a riding model approximation. The (-3 - 2 1) and (-4 - 3 1) reflections were omitted owing to bad disagreement.

### Crystal data

**Table:**

C_15_H_13_N_5_O	*Z* = 2
*M**_r_* = 279.30	*F*(000) = 292
Triclinic, *P*1	*D*_x_ = 1.379 Mg m^−^^3^
Hall symbol: -P 1	Mo *K*α radiation, λ = 0.71073 Å
*a* = 8.633 (2) Å	Cell parameters from 3872 reflections
*b* = 8.763 (3) Å	θ = 3.1–27.5°
*c* = 9.559 (3) Å	µ = 0.09 mm^−^^1^
α = 91.715 (7)°	*T* = 120 K
β = 108.110 (8)°	Cut Block, colourless
γ = 100.572 (7)°	0.52 × 0.44 × 0.18 mm
*V* = 672.7 (4) Å^3^	

### Data collection

**Table:**

Rigaku R-AXIS conversion diffractometer	3067 independent reflections
Radiation source: Sealed Tube	1884 reflections with *I* > 2σ(*I*)
Graphite Monochromator monochromator	*R*_int_ = 0.081
Detector resolution: 10.0000 pixels mm^-1^	θ_max_ = 27.5°, θ_min_ = 3.1°
profile data from ω–scans	*h* = −11→11
Absorption correction: multi-scan (*CrystalClear*-SM Expert; Rigaku, 2011)	*k* = −11→11
*T*_min_ = 0.953, *T*_max_ = 0.984	*l* = −12→12
8461 measured reflections	

### Refinement

**Table:**

Refinement on *F*^2^	Primary atom site location: structure-invariant direct methods
Least-squares matrix: full	Secondary atom site location: difference Fourier map
*R*[*F*^2^ > 2σ(*F*^2^)] = 0.075	Hydrogen site location: inferred from neighbouring sites
*wR*(*F*^2^) = 0.225	H-atom parameters constrained
*S* = 1.05	*w* = 1/[σ^2^(*F*_o_^2^) + (0.1134*P*)^2^] where *P* = (*F*_o_^2^ + 2*F*_c_^2^)/3
3067 reflections	(Δ/σ)_max_ < 0.001
190 parameters	Δρ_max_ = 0.35 e Å^−^^3^
0 restraints	Δρ_min_ = −0.39 e Å^−^^3^

### Special details

**Table:**

Experimental. Rigaku *CrystalClear*-SM Expert 2.0 r10
Geometry. Bond distances, angles *etc*. have been calculated using the rounded fractional coordinates. All su's are estimated from the variances of the (full) variance-covariance matrix. The cell e.s.d.'s are taken into account in the estimation of distances, angles and torsion angles
Refinement. Refinement on *F*^2^ for ALL reflections except those flagged by the user for potential systematic errors. Weighted *R*-factors *wR* and all goodnesses of fit *S* are based on *F*^2^, conventional *R*-factors *R* are based on *F*, with *F* set to zero for negative *F*^2^. The observed criterion of *F*^2^ > σ(*F*^2^) is used only for calculating -*R*-factor-obs *etc*. and is not relevant to the choice of reflections for refinement. *R*-factors based on *F*^2^ are statistically about twice as large as those based on *F*, and *R*-factors based on ALL data will be even larger.

### Fractional atomic coordinates and isotropic or equivalent isotropic displacement parameters (Å^2^)

**Table:**

	*x*	*y*	*z*	*U*_iso_*/*U*_eq_	
O1	0.3867 (2)	0.3040 (2)	−0.00807 (19)	0.0420 (6)	
N1	0.4420 (3)	0.2960 (2)	0.4639 (2)	0.0340 (7)	
N2	0.5601 (3)	0.1672 (2)	0.6628 (2)	0.0343 (7)	
N3	0.3463 (3)	0.4202 (3)	0.2558 (2)	0.0362 (7)	
N4	0.1904 (3)	−0.0380 (3)	0.7594 (2)	0.0435 (8)	
N5	−0.0679 (3)	0.4902 (3)	0.2845 (3)	0.0481 (9)	
C1	0.4195 (3)	0.2145 (3)	0.5715 (3)	0.0315 (8)	
C2	0.2626 (3)	0.1732 (3)	0.5944 (3)	0.0332 (8)	
C3	0.1383 (3)	0.2445 (3)	0.5105 (3)	0.0362 (8)	
C4	0.1611 (3)	0.3373 (3)	0.4010 (3)	0.0341 (8)	
C5	0.3158 (3)	0.3500 (3)	0.3710 (3)	0.0327 (8)	
C6	0.2270 (3)	0.0584 (3)	0.6897 (3)	0.0368 (8)	
C7	0.0352 (3)	0.4214 (3)	0.3333 (3)	0.0383 (9)	
C8	0.5972 (3)	0.1465 (3)	0.8128 (3)	0.0363 (8)	
C9	0.7503 (3)	0.1120 (3)	0.8612 (3)	0.0395 (9)	
C10	0.8110 (3)	0.1098 (3)	0.7379 (3)	0.0405 (9)	
C11	0.6950 (3)	0.1461 (3)	0.6203 (3)	0.0385 (9)	
C12	0.5130 (3)	0.4356 (3)	0.2379 (3)	0.0366 (8)	
C13	0.5100 (3)	0.3023 (3)	0.1320 (3)	0.0383 (8)	
C14	0.2248 (3)	0.2914 (3)	0.0054 (3)	0.0389 (9)	
C15	0.2197 (3)	0.4214 (3)	0.1118 (3)	0.0384 (8)	
H3	0.03690	0.22960	0.52830	0.0430*	
H8	0.52860	0.15490	0.86970	0.0440*	
H9	0.80610	0.09320	0.95720	0.0470*	
H10	0.91250	0.08720	0.73900	0.0490*	
H11	0.70370	0.15550	0.52630	0.0460*	
H12A	0.59600	0.43340	0.33300	0.0440*	
H12B	0.54200	0.53420	0.19960	0.0440*	
H13A	0.61850	0.31190	0.11930	0.0460*	
H13B	0.48530	0.20400	0.17250	0.0460*	
H14A	0.19270	0.19150	0.04040	0.0470*	
H14B	0.14510	0.29540	−0.09100	0.0470*	
H15A	0.24120	0.52120	0.07280	0.0460*	
H15B	0.11030	0.40650	0.12330	0.0460*	

### Atomic displacement parameters (Å^2^)

**Table:**

	*U*^11^	*U*^22^	*U*^33^	*U*^12^	*U*^13^	*U*^23^
O1	0.0463 (11)	0.0543 (12)	0.0264 (9)	0.0118 (9)	0.0128 (9)	−0.0030 (8)
N1	0.0395 (12)	0.0407 (12)	0.0237 (11)	0.0128 (10)	0.0107 (10)	−0.0010 (9)
N2	0.0386 (12)	0.0425 (12)	0.0267 (11)	0.0155 (10)	0.0137 (10)	0.0010 (9)
N3	0.0412 (12)	0.0444 (13)	0.0252 (11)	0.0124 (10)	0.0120 (10)	0.0022 (9)
N4	0.0461 (13)	0.0517 (15)	0.0361 (13)	0.0124 (12)	0.0164 (11)	0.0070 (11)
N5	0.0503 (14)	0.0567 (16)	0.0453 (15)	0.0213 (13)	0.0207 (12)	0.0065 (12)
C1	0.0359 (13)	0.0348 (14)	0.0255 (12)	0.0098 (11)	0.0110 (11)	−0.0015 (10)
C2	0.0389 (14)	0.0374 (14)	0.0261 (13)	0.0102 (12)	0.0133 (11)	−0.0007 (10)
C3	0.0390 (14)	0.0423 (15)	0.0292 (13)	0.0117 (12)	0.0123 (12)	−0.0044 (11)
C4	0.0366 (14)	0.0396 (14)	0.0269 (13)	0.0115 (12)	0.0095 (12)	−0.0009 (11)
C5	0.0391 (14)	0.0356 (14)	0.0241 (12)	0.0082 (12)	0.0111 (11)	−0.0015 (10)
C6	0.0383 (14)	0.0463 (16)	0.0282 (13)	0.0149 (13)	0.0112 (12)	−0.0025 (11)
C7	0.0435 (15)	0.0468 (16)	0.0297 (14)	0.0150 (14)	0.0158 (13)	0.0028 (12)
C8	0.0464 (15)	0.0375 (14)	0.0277 (13)	0.0108 (12)	0.0145 (12)	0.0028 (10)
C9	0.0452 (15)	0.0417 (16)	0.0298 (14)	0.0131 (13)	0.0071 (12)	0.0012 (11)
C10	0.0390 (14)	0.0489 (17)	0.0372 (15)	0.0154 (13)	0.0134 (13)	0.0055 (12)
C11	0.0400 (14)	0.0492 (17)	0.0314 (14)	0.0169 (13)	0.0143 (12)	0.0010 (11)
C12	0.0412 (14)	0.0439 (15)	0.0245 (12)	0.0082 (12)	0.0107 (12)	0.0016 (11)
C13	0.0403 (14)	0.0460 (16)	0.0298 (13)	0.0114 (13)	0.0119 (12)	0.0004 (11)
C14	0.0425 (15)	0.0460 (16)	0.0290 (14)	0.0110 (13)	0.0117 (12)	0.0009 (11)
C15	0.0456 (15)	0.0459 (15)	0.0252 (13)	0.0155 (13)	0.0101 (12)	0.0030 (11)

### Geometric parameters (Å, º)

**Table:**

O1—C13	1.431 (3)	C8—C9	1.352 (4)
O1—C14	1.428 (3)	C9—C10	1.432 (4)
N1—C1	1.313 (3)	C10—C11	1.344 (4)
N1—C5	1.346 (4)	C12—C13	1.514 (4)
N2—C1	1.398 (4)	C14—C15	1.520 (4)
N2—C8	1.394 (3)	C3—H3	0.9300
N2—C11	1.387 (4)	C8—H8	0.9300
N3—C5	1.350 (3)	C9—H9	0.9300
N3—C12	1.484 (4)	C10—H10	0.9300
N3—C15	1.469 (3)	C11—H11	0.9300
N4—C6	1.151 (4)	C12—H12A	0.9700
N5—C7	1.153 (4)	C12—H12B	0.9700
C1—C2	1.422 (4)	C13—H13A	0.9700
C2—C3	1.388 (4)	C13—H13B	0.9700
C2—C6	1.432 (4)	C14—H14A	0.9700
C3—C4	1.384 (4)	C14—H14B	0.9700
C4—C5	1.437 (4)	C15—H15A	0.9700
C4—C7	1.424 (4)	C15—H15B	0.9700
			
C13—O1—C14	111.61 (19)	C2—C3—H3	119.00
C1—N1—C5	120.8 (3)	C4—C3—H3	119.00
C1—N2—C8	127.1 (2)	N2—C8—H8	126.00
C1—N2—C11	124.7 (2)	C9—C8—H8	126.00
C8—N2—C11	108.1 (2)	C8—C9—H9	126.00
C5—N3—C12	119.8 (2)	C10—C9—H9	126.00
C5—N3—C15	124.4 (2)	C9—C10—H10	126.00
C12—N3—C15	110.1 (2)	C11—C10—H10	126.00
N1—C1—N2	115.8 (2)	N2—C11—H11	126.00
N1—C1—C2	123.1 (3)	C10—C11—H11	126.00
N2—C1—C2	121.2 (2)	N3—C12—H12A	110.00
C1—C2—C3	115.8 (2)	N3—C12—H12B	110.00
C1—C2—C6	123.7 (2)	C13—C12—H12A	110.00
C3—C2—C6	120.4 (3)	C13—C12—H12B	110.00
C2—C3—C4	121.8 (3)	H12A—C12—H12B	108.00
C3—C4—C5	117.3 (2)	O1—C13—H13A	110.00
C3—C4—C7	117.9 (3)	O1—C13—H13B	110.00
C5—C4—C7	124.6 (2)	C12—C13—H13A	110.00
N1—C5—N3	116.6 (3)	C12—C13—H13B	110.00
N1—C5—C4	119.9 (2)	H13A—C13—H13B	108.00
N3—C5—C4	123.5 (3)	O1—C14—H14A	109.00
N4—C6—C2	176.0 (3)	O1—C14—H14B	109.00
N5—C7—C4	176.9 (3)	C15—C14—H14A	109.00
N2—C8—C9	108.1 (2)	C15—C14—H14B	109.00
C8—C9—C10	107.6 (2)	H14A—C14—H14B	108.00
C9—C10—C11	107.8 (2)	N3—C15—H15A	110.00
N2—C11—C10	108.5 (2)	N3—C15—H15B	110.00
N3—C12—C13	109.2 (2)	C14—C15—H15A	110.00
O1—C13—C12	110.1 (2)	C14—C15—H15B	110.00
O1—C14—C15	111.6 (2)	H15A—C15—H15B	108.00
N3—C15—C14	109.3 (2)		
			
C14—O1—C13—C12	−58.8 (3)	C5—N3—C15—C14	−96.4 (3)
C13—O1—C14—C15	57.6 (3)	C12—N3—C5—N1	−0.5 (4)
C1—N1—C5—C4	8.6 (4)	N2—C1—C2—C6	−12.6 (4)
C5—N1—C1—N2	−178.8 (2)	N2—C1—C2—C3	171.4 (2)
C5—N1—C1—C2	2.3 (4)	N1—C1—C2—C3	−9.8 (4)
C1—N1—C5—N3	−174.1 (2)	N1—C1—C2—C6	166.3 (2)
C8—N2—C1—C2	−32.7 (4)	C6—C2—C3—C4	−169.8 (3)
C1—N2—C11—C10	176.3 (2)	C1—C2—C3—C4	6.3 (4)
C8—N2—C1—N1	148.3 (2)	C2—C3—C4—C7	−172.3 (3)
C1—N2—C8—C9	−175.3 (2)	C2—C3—C4—C5	3.6 (4)
C11—N2—C8—C9	−0.6 (3)	C3—C4—C5—N1	−11.4 (4)
C8—N2—C11—C10	1.4 (3)	C7—C4—C5—N3	−13.0 (4)
C11—N2—C1—C2	153.4 (2)	C7—C4—C5—N1	164.2 (2)
C11—N2—C1—N1	−25.6 (3)	C3—C4—C5—N3	171.5 (3)
C12—N3—C15—C14	56.8 (3)	N2—C8—C9—C10	−0.4 (3)
C15—N3—C5—C4	−32.6 (4)	C8—C9—C10—C11	1.3 (3)
C5—N3—C12—C13	95.8 (3)	C9—C10—C11—N2	−1.7 (3)
C15—N3—C12—C13	−58.8 (3)	N3—C12—C13—O1	59.0 (3)
C15—N3—C5—N1	150.2 (2)	O1—C14—C15—N3	−56.1 (3)
C12—N3—C5—C4	176.7 (2)		

### Hydrogen-bond geometry (Å, º)

Cg3 is the centroid of the N1/C1–C5 pyridine ring.

**Table:**

*D*—H···*A*	*D*—H	H···*A*	*D*···*A*	*D*—H···*A*
C8—H8···O1^i^	0.93	2.44	3.294 (3)	152
C12—H12*A*···N1	0.97	2.31	2.692 (3)	103
C12—H12*B*···O1^ii^	0.97	2.51	3.397 (3)	153
C12—H12*A*···*Cg*3^iii^	0.97	2.92	3.429 (3)	114

Symmetry codes: (i) *x*, *y*, *z*+1; (ii) −*x*+1, −*y*+1, −*z*; (iii) −*x*+1, −*y*+1, −*z*+1.
